# Total Antioxidant Capacity of *Arachis hypogaea* Seed Kernels and Coats: An Analytical and Sensory Investigation

**DOI:** 10.3390/ijms26135990

**Published:** 2025-06-22

**Authors:** Julie Marshall, Lissa Gilliam, Melanie McGilton, Ana Patty, Lily Sowell, Ashley Cherry, Brian Fisher, Matt Scholten, Chris Liebold, Darlene Cowart, Samara Sterling

**Affiliations:** 1 Lubbock Christian University, 5601 19th ST, Lubbock, TX 79407, USA; lissa.gilliam@lcu.edu (L.G.); melanie.mcgilton@lcu.edu (M.M.); apatty4424@lcu.edu (A.P.); lsowell2485@lcu.edu (L.S.); ashley.cherry@lcu.edu (A.C.); brian.fisher@lcu.edu (B.F.); 2 The J.M. Smucker Co., Orrville, OH 444667, USA; matt.scholten@jmsmucker.com (M.S.); chris.liebold@jmsmucker.com (C.L.); 3 Birdsong Peanuts, Suffolk, VA 23434, USA; dcowart@birdsongpeanuts.com; 4 The Peanut Institute, Albany, GA 31708, USA; ssterling@peanut-institute.com

**Keywords:** peanut flavor, catechins, stilbenes, flavonoids, phenolic acids

## Abstract

Antioxidants are critical components of the body’s defense system, providing protection against cell-damaging free radicals responsible for oxidative damage of biomolecules. Humans benefit from the consumption of plants with high antioxidant content, which have been shown to positively impact health. In plant physiology, antioxidants provide protection from biotic and abiotic stress, particularly during the development of seeds and germination. Peanut seeds and seed coats have been shown to contain several beneficial antioxidants and are a good source of phytonutrients. Seed coat color can vary greatly and impact the antioxidant capacity of the edible portion of the peanut. Additionally, the seed coat can provide bitter notes in products, affecting their palatability and potentially negating the beneficial properties of the antioxidants present. A total of 42 accessions from the Germplasm Resource Information Network (GRIN) with a variety of seed coat colors were obtained and analyzed for total antioxidant capacity to provide a baseline assessment of the distribution of antioxidants in kernel versus seed coats. The results demonstrated that seed coat color somewhat impacts antioxidant capacity, and 56–88% of the total antioxidant capacity resides in the seed kernel. Three control samples, not part of the germplasm collection, were roasted and prepared for analysis by the descriptive sensory panel. Seed coats were added back to the roasted paste in increasing proportion for analysis by the panel, and perceptions regarding bitterness and overall organoleptic properties were noted. Based on the results of this study, several accessions were selected and then planted for increase and potential crossbreeding with appropriate commercial cultivars. This information could be used to selectively add antioxidant capacity to peanut breeding programs to provide additional health benefits to consumers without compromising the sensory perception and desirability and peanut products in nutrition.

## 1. Introduction

The nutritional composition of peanuts is approximately 50% fat, 25% protein, and 8.4% fiber [1]. Regular consumption of peanuts has been shown to decrease cardiovascular disease risk by 50%, as well as decrease blood pressure and inflammation [2]. Peanuts have not only been shown to reduce the risk of type 2 diabetes but have also been shown to help people with type 2 diabetes regulate blood sugar levels. Despite the elevated level of fat in peanuts, it has been shown through numerous studies that a diet including peanuts does not correlate to a higher BMI (Body Mass Index) but does, however, correlate to a higher quality of food intake [3,4]. In a randomized control study, two groups of participants at risk for type 2 diabetes were advised to maintain a calorie-deficit diet. Researchers advised the control group to abstain from peanut products and advised the treatment group to consume 35 g of salted peanuts twice daily, before meals. The results showed no discernable difference in weight loss between the control and treatment groups but did observe a 10% risk decrease in a future cardiovascular event in the treatment group.

The high-fat concentration of peanuts designates the *Arachis hypogea* plant as an oilseed crop. Oilseeds represent a versatile crop that is grown for multiple industries including oleochemical, agricultural, and food industries [5]. Peanuts, in particular, support various industries depending on what organ of the plant is used. The kernel is the largest tissue of the peanut, which comprises the majority of what consumers eat.

Peanuts provide a host of antioxidants that can positively impact human health. Regular inclusion of peanuts in the dietary pattern can provide consumers with functionality beyond basic nutrition to prevent chronic disease and promote general health and wellness.

Peanuts contain a wide variety of compounds that have been shown to decrease oxidative stress and lower the risk of chronic inflammatory diseases including cardiovascular disease, diabetes, hypertension, and cancer. One meta-analysis found that a diet containing 25 g of peanuts per day may help lower both total cholesterol and LDL, as well as blood triglycerides, in addition to lowering the risk of cardiovascular disease by 13% [6]. Furthermore, the American Diabetes Association lists legumes, including peanuts, as a “diabetes superfood” due to their low glycemic index and high dietary fiber; because they contain all 20 amino acids and a high proportion of monounsaturated fatty acids, such as oleic acid, they are also a popular choice in plant-based diets [7]. A diet containing fatty peanut products was found to be more effective than a low-fat diet in lowering total cholesterol and the LDL/HDL ratio [8]. However, many of peanuts’ most notable health benefits stem from their antioxidant content, including phenolic compounds, flavonoids, anthocyanidins and proanthocyanidins, and bioactive peptides and enzymes. Peanuts, especially peanut skins, also referred to as seed coats, contain a high concentration of compounds with ROS (reactive oxygen species)-scavenging abilities. The treatment of liver cells with peanut extract was found to reduce ROS content by up to 38%, as well as prevent the upregulation of several antioxidant enzymes and reduce the production of nitric oxide [9].

Peanuts are a legume crop grown worldwide across six continents. Peanuts have a high fat content of around 50% and are, therefore, categorized as oilseeds. Agriculturally, peanuts are categorized as oilseeds and can be compared to sunflower seeds, rapeseed, and sesame seeds. Every year, the peanut crop produces an average of 45 million metric tons worldwide. USDA-FAS reported a 3.57 million metric ton increase in peanut production from 2019 to 2024 [10]. Every year, the United States exports an average of over 500,000 metric tons valued at over USD 675 million [11]. Most recently, in 2024, the value of peanuts exported from the United States rose to over USD 722 million [12]. Peanuts represent a valuable food commodity for a changing climate, as the water footprint of peanut protein is lower than that of all other common protein sources (14.6 L/g ground nut protein vs. 65.4 L/g almond protein and 30.4 L/g chicken protein) [13]. Water footprint is a metric used to determine the amount of water needed to grow a standard amount of crop to fruition.

Due to their low water footprint, peanuts are grown globally across several drought-prone regions. Much of this growth occurs in Asia, Africa, South America, and North America, although peanut agriculture is found globally in over 100 countries [14]. The global prevalence of peanut agriculture has led to their inclusion in a wide array of cuisines and cultures. Peanuts are consumed in raw, roasted, and boiled forms. They are sold for consumption at all stages of processing including in-shell, shelled raw and roasted, or processed further into peanut paste or meal [8]. Globally, peanuts are known as a vital source of nutrition for malnourished communities, and the influence of products, such as PlumpyNut™, and other ready-to-use therapeutic foods have had a major influence in hunger-stricken regions [15].

The impact of antioxidants on human health is still being investigated. However, antioxidants have long been known to play a key role in neutralizing free radicals that cause oxidative damage to DNA, proteins, and lipids [16]. Extensive oxidative damage can lead to premature aging and certain conditions like cardiovascular disease, cancer, and neurodegenerative diseases [16]. Certain antioxidants may also block secondary mechanisms that contribute to inflammatory pathways involved in gut health and immunity [17,18]. Some antioxidant compounds are further involved in potential tumor suppression through cell cycle arrest and apoptosis [19,20]. While the bioavailability, bioaccessibility, and absorption of various antioxidants should be considered in their application in human health [21], long-term consumption of diets rich in antioxidants appears to offer protection against the development of cancers, cardiovascular diseases, diabetes, osteoporosis, and neurodegenerative diseases in humans [16].

Antioxidants, including flavonoids and other phenolic compounds, are often produced in response to biotic and abiotic stresses in plants as a method of protection from damage. Post-harvest stress to peanut kernels (mechanical damage, such as slicing or crushing) may increase resveratrol content [22]. Elevated ambient ozone levels during plant growth increase antioxidant capacity in peanut plants, as well as improve the plants’ ability to respond to other oxidative stresses later in life [23]. Drought stress, which is known to cause an increase in oxidative stress and, consequently, alters plants’ proteomes and gene expression, upregulates the production of many phenolic compounds and antioxidant enzymes, resulting in a commensurate increase in antioxidant capacity [24,25,26].

Fungal infections of food and feed crops, especially those caused by *Aspergillus flavus* and *Aspergillus parasiticus*, can result in the accumulation of aflatoxins, a carcinogenic and potentially deadly contaminant [27,28]. These infections are even more common in crops grown under drought conditions in arid and semi-arid regions. A correlation between peanut kernel phenolic content and resistance to aflatoxin production has been discovered [29]. Fungal stress has been observed to upregulate the production of several phytoalexins, including resveratrol; additionally, ROS-scavenging flavonoids, such as apigenin and quercetin, damage the walls of A. flavus cells and inhibit fungal growth [30].

The variations and cultivars of Arachis hypogea have been shown to vary in micronutrient concentrations but have a similarity in macronutrients. Peanut skins comprise approximately 38.8–42.2% fiber, 8.88–12.7% protein, 9.59–10.2% fat, and 2.07–2.13% ash [31]. Though the peanut skin only contributes 3% of the peanut kernels’ unblanched mass, the fiber and bioactive compounds contained within make them valuable commodities as functional food [31]. Additionally, several studies have revealed the high total phenolic content of peanut skins. These powerful polyphenols have antioxidant and cholesterol-lowering properties.

Antioxidants found in plants typically take one of two forms, small-molecule phenolic compounds or antioxidant enzymes, of which phenolic compounds are the more bioavailable [32]. In peanuts, most of the antioxidant phenolic compounds are concentrated in the skins of the nuts, where they serve to protect the nut from external oxidative damage. After germination, however, antioxidant content in skins decreases, while antioxidant content in kernels increases as the peanut shifts to protect the growing plant [33]. Phenolic pigments, such as proanthocyanins, give peanut skins their dark red-brown color, with darker peanut skins typically having a higher antioxidant capacity than lighter ones. Beyond the skin, peanut kernels and hearts typically have the same phenolic composition regardless of skin color; resveratrol, epicatechin, quercetin, and phenolic acids including p-hydroxybenzoic, coumaric, and sinapic acid are common [34]. The peanut heart refers to the embryonic root of the seed. One study found that up to 88% of phenolics in peanuts are found in the hydrophilic (i.e., non-lipid) portion of the nuts. Furthermore, evidence suggests that studies utilizing solvent-based extractions may drastically under-report the total phenolic content (TPC) of peanuts compared to studies utilizing hydrolysis extractions [35]. This suggests that a large portion of peanut antioxidants may be present in a bound form; while this fraction of the TPC has previously been thought to be poorly bioavailable and, therefore, ignorable, more research is needed.

Figures S1–S4 in the Supplementary Materials were created from recently published studies compiling a list of 109 antioxidants found in peanut skin alongside the method that was used for identification. Compounds have been categorized as catechins and proanthocyanidins, stilbenes, flavonoids, and free phenolic acids and esters.

Researchers have recently begun to focus on the valorization of peanut skins. Several studies have looked at the various ways in which peanut skins may be utilized. In 2020, *Applied Science* published a review of the various applications of peanut skins and peanut skin extract. Due to the tannins present, peanut skins have been shown to have chelation properties useful to the wastewater industry [36]. In vitro applications utilizing peanut skin extract have been shown to prolong the shelf life of raw meats, including ground beef and chicken, giving support to the antioxidant and antimicrobial properties of peanut skins [37]. Further antimicrobial properties were observed when apple juice was supplemented with peanut skin extract. *Saccharomyces cerevisiae*, *Zygosaccharomyces bailii*, and *Zygosaccharomyces bisporus* yeasts were inhibited in apple juice for 120 h when supplemented with peanut skin extract [38]. Further studies of peanut skins have been used to increase the antioxidant properties of regularly consumed ready-to-eat foods. Carmago et al. supplemented an oatmeal chocolate chip cookie with peanut skins up to 2.5% and recorded an increase in total polyphenols, antioxidant capacity, and insoluble fiber and a decrease in carbohydrates, with no discernable change in sensory experience [39]. Using peanut skin extract powder supplementation in buffalo yogurt (50 mg/L), Hamad et al. observed an increase in total antioxidant capacity (TAC) and total phenolic content (TPC) while decreasing whey separation and maintaining consumer acceptability [40].

The increased phenolic content of peanut-skin-enriched peanut butter has been documented along with favorable sensory outcomes in multiple applications. Ma et al. performed a ferric-ion-reducing antioxidant power (FRAP) assay on peanut butter samples supplemented with peanut skins at 1.25%, 2.5%, 3.75%, and 5.0% formulations and observed an antioxidant power increase of 86%, 357%, 533%, and 714%, respectively. In a similar study, peanut butter formulated with increasing percentages of peanut skin was tested for sensory experience. In this study, the panelists determined the flavor markers were unchanged at 2.5% peanut skins (PSs). The 5% PS formulation had decreases in flavor acceptability and texture markers, such as gumminess [41,42].

In vivo animal studies have also shown promise for the utilization of peanut skins in human health [36]. Mice-based studies have shown that when fed high-fat diets supplemented with peanut skin extract, a decrease in cholesterol in the liver and glycogen is observed. Additionally, a methanol peanut skin extraction given to mice prior to a chloroform injection was shown to protect the liver from chloroform-induced damage. An animal-based study with rat subjects centered on the supplementation of a high-fat diet with peanut skin extract and saw a decrease in blood lipids and an increase in the bioavailability of the antioxidant proanthocyanidin A2 [36].

The value of adding peanut skins/seed coats to food applications for enhanced antioxidant capacity (AOC) is compelling. Additionally, the diversity of seed coat color available within the peanut germplasm and proven potential as a source of healthful AOC provides an opportunity to potentially increase the antioxidant content of peanuts overall, benefiting the health of the plant as well as the consumer. To investigate the possibility of increasing the AOC of peanuts, several questions must be answered, including identifying the diversity of seed coat color in germplasm resources, determining the proportion of AOC in the kernel versus seed coat, optimizing an assay to allow comparisons between samples that accurately reflect the bioavailability of the nutrients in physiological systems, and assessing the impact of adding seed coats to peanut products from both a consumer acceptance aspect as well as providing a significant increase in the AOC of the food source. To accomplish these aims, peanut samples with a variety of seed coat colors were gathered and assayed for total antioxidant capacity, and seed coats were added to roasted peanut paste to assess the total antioxidant capacity as well as the palatability of the seed coat-enhanced food. The proportion of AOC in the skin versus kernel, the impact of seed coat color on TAC, the sensory results from descriptive flavor analysis, and the TAC of peanut paste with skin supplementation are deemed to be statistically significant and are reported to identify germplasm for selective breeding and opportunities for the improvement of TAC for the added health benefits to consumers.

## 2. Results

**Figure 1:**
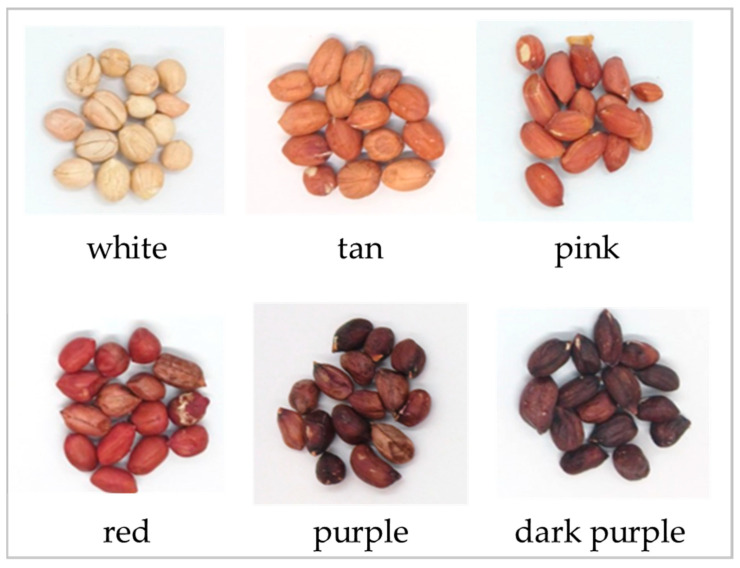
Six different seed color descriptions were identified in the sample set. Seed color descriptions were determined from the GRIN database.

A total of 54 GRIN accessions were selected for analysis based on their skin color and maturation rate as listed in the GRIN database (Figure 1 and Figure S7a–e). The percent skin content for each individual sample was calculated by removing the seed coat from five separate kernels and calculating the mass of the total. Using a scalpel, the skin and kernel fractions were cut into small pieces to be massed as separate fractions for total antioxidant assay. For the initial twelve accessions, each sample was analyzed in duplicate. For the remaining forty-two samples, the samples were assayed singly. For all samples, replicate wells were run on the assay plate and averaged. The accuracy and precision of the assay were calculated as an STD of 0.037 with a % coefficient of variation of 8.80.

**Figure 2:**
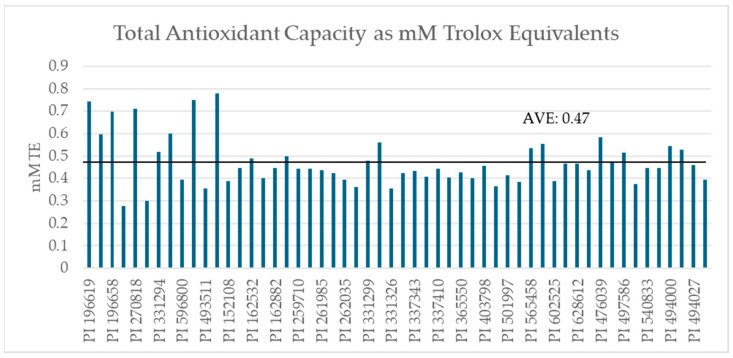
Mass-adjusted Total Antioxidant Capacity of GRIN samples (n = 54) expressed as mM Trolox equivalents as determined by commercial CuPRAC assay.

The average mass-adjusted TAC of the set was 0.47 mM TE. The data showed that 56.3 to 88.4% of TAC was contained in the kernels, with an average of 73.9% (Table 1). Skins ranged from 0.05 to 0.25 mM TE and kernels ranged from 0.20 to 0.68 mM TE after mass adjustment (Figure 2).

**Figure 3:**
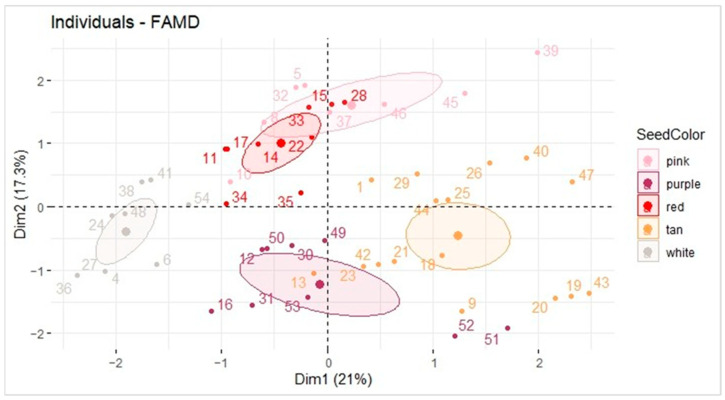
FAMD plot of seed coat color, TAC, and market type (Runner, Spanish, Valencia, and Virginia). Data generated using the sample set referenced in Table 1.

Seed coat color, market type, and TAC were evaluated using a factor analysis of mixed data (FAMD). TAC is the only quantitative variable, so the FAMD was performed to seek the relationship between quantitative data (TAC) and qualitative data (seed color and type) (Figure 3).

The white seed coat color samples seemed to be inversely correlated with TAC, as an FAMD plot of the variables showed that TAC was pointing along the x-axis in quadrant I, and the white peanuts were clustered 180° in the opposite direction near the x-axis. The percentage explained variance represented by the graph, including the three variables, sums to 38.3%. This is not ideal, and the results suggest that darker seed coats do not equate to higher TAC despite the clustering of the white coat samples. For this reason, other methods were used to compare the market types, seed coat colors, and TAC.

**Figure 4:**
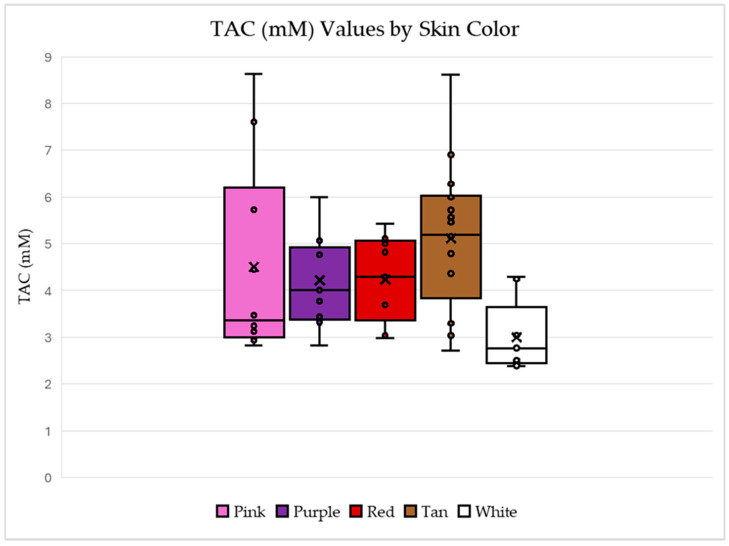
Box-and-whisker plots for the measured TAC values for each skin color which include each marked data point and an “x” to represent the mean. These plots visually represent the five-number summary for the data (the maximum, minimum, and quartiles) and show both the range of the data and the inner 50% of the data for each plot. Data generated using the sample set referenced in Table 1.

**Figure 5:**
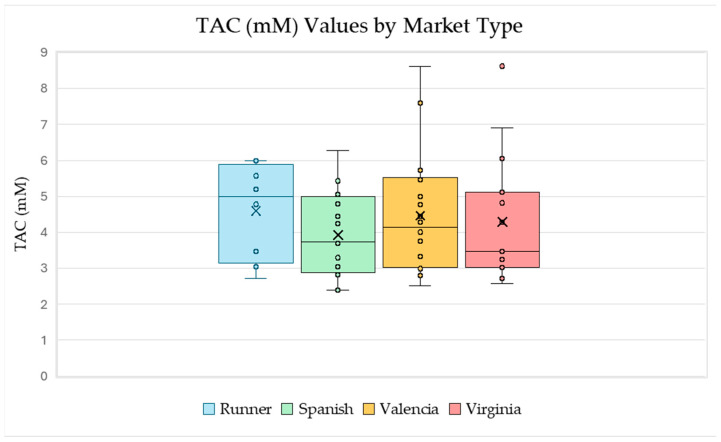
Box-and-whisker plots for the measured TAC values for each market type which include each marked data point and an “x” to represent the mean. These plots visually represent the five-number summary for the data (the maximum, minimum, and quartiles) and show both the range of the data and the inner 50% of the data for each plot. Several samples in our data set were a combination of two market types, in which case the measured TAC value was included in both box-and-whisker plots. Data generated using the sample set referenced in Table 1.

Figure 4 shows the relationship between TAC first as compared to seed coat colors and then market type in Figure 5. For the box and whisker plot of TAC and skin color, the largest individual TAC values are from the samples with pink or tan skin colors. The white seed coat color has the smallest TAC values and the tan has the largest average and median TAC values.

These results are not expected, as brightly colored fruits (including skins) and vegetables are known to possess high antioxidant capacity. The results suggest that specific antioxidant compound composition in peanuts is not necessarily correlated with dark purple and pink color pigmentation.

Next, TAC was compared to peanut market types Runner, Spanish, Valencia, and Virginia (Figure 5). The largest individual TAC values were of the Valencia and Virginia market types. The Runner market type has the largest average and median TAC values. This might be caused by the fact that there were no white seed coat samples among the selected Runner samples. Additionally, the white seed coat set tended to have the lowest TAC values across all market types, as shown in Figure 4.

**Figure 6:**
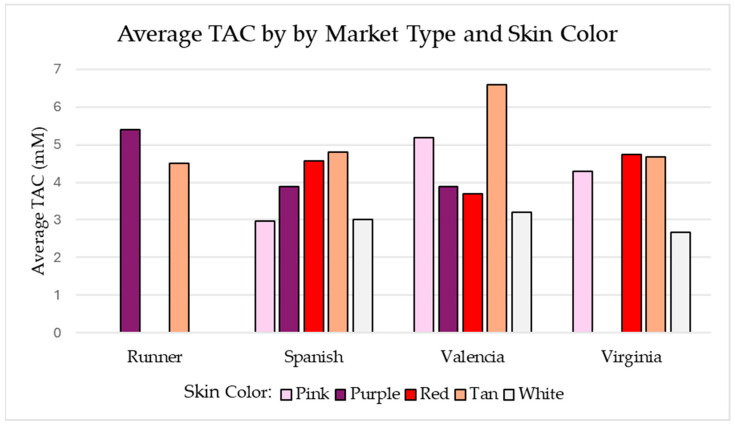
Shows the average TAC (mM) for each market type-skin color combination with at least two measured samples. Data generated using the sample set referenced in Table 1.

The average TAC by market type and seed coat color is shown in the following set of bar graphs in Figure 6. The Runner market type had less variation in seed coat colors available in the collection. White seed coat color averaged the lowest TAC values across the board, except for the Spanish market type in which the white and pink seed coat colors were comparable. The tan Valencia sample set had the largest TAC values of all market type–seed coat color combinations. All three tan Valencia samples had a TAC value greater than 5.45 mM, which is larger than the average of any other market type–seed coat color combinations.

Southeast Jumbo Runner peanuts were pre-dried or roasted following the Sensory Paste Preparation Protocol of Biochemistry Research Lab Analytical Services. A composite sample (five red-skinned, unblanched peanuts pre-dried and roasted) was selected and dissected into kernels, hearts, and skins. Pre-dried and roasted peanuts were then blanched or kept red-skinned and processed into rough meals and pastes. Roasted paste samples were supplemented with peanut skins at 0%, 1%, 3%, and 5%. A red-skinned paste made from unblanched, roasted peanuts, and a commercially available “Unblanched, Unsalted Peanut Butter” were also included. All roasted paste samples were sent to an expert sensory panel at Lubbock Christian University and followed sensory tasting protocols of the Biochemistry Research Lab. Sensory panelists were compensated for their time by the Biochemistry Research Lab. All kernels, hearts, skins, meals, and pastes were tested for total antioxidant capacity using the total antioxidant capacity assay kit supplied by Abcam (ab65329) with a modified protocol. This method and kit were selected for the applicability of the Cupric ion-reducing antioxidant capacity (CuPRAC) assay to food.

The roasted paste samples were sent to an expert sensory panel (*n* = 5) and analyzed on a modified 10-point flavor profile, a hedonic ranking, and a line scale ranking of appearance, mouthfeel, and overall impression. The 10-point flavor lexicon of peanuts defined by Johnsen et al. was used to properly analyze the flavor of peanuts [43]. Flavor descriptors included “Roast Peanut” (RP), “Sweet”, “Sweet Aromatic” (SA), “Salty”, “Bitter”, and “Raw/Bean/Green” (RBG), “Coffee/Dark Roast” (C/DR), “Woody/Hull/Skins” (WHS), and “Astringent” (AST). In this study, due to the uniformity of peanut type, source, and roasting treatment, the flavor signifiers that suggest a poor-quality peanut were excluded. Excluded descriptors included “Fruity, Fermented, Sour, or Overripe” (FSOR); Cardboard (CBDB); and Paint/Old Oil (POO)”. A preference ranking test was used to determine the preference of pastes from most preferred to least preferred. Panelists were asked to rank samples according to preference, with “1” being most preferred and “6” being least preferred. A hedonic structured line scale was used to determine overall likeability in the following categories: appearance, mouthfeel, and overall impression [44]. For each sample, panelists were asked to draw a line perpendicular to the printed line designating where they would place the value according to each category. Sensory panelists had been previously trained in peanut lexicon standards by undergoing intensive initial training and sustained, monitored training for a period of 3–6 months prior to being allowed to participate in the panel. The testing protocol used was supplied by the Biochemistry Research Lab at Lubbock Christian University. Panelists recorded their responses on paper ballots supplied (Figures S5 and S6).

### 2.1. TAC of Anatomic Peanut Parts

**Figure 7:**
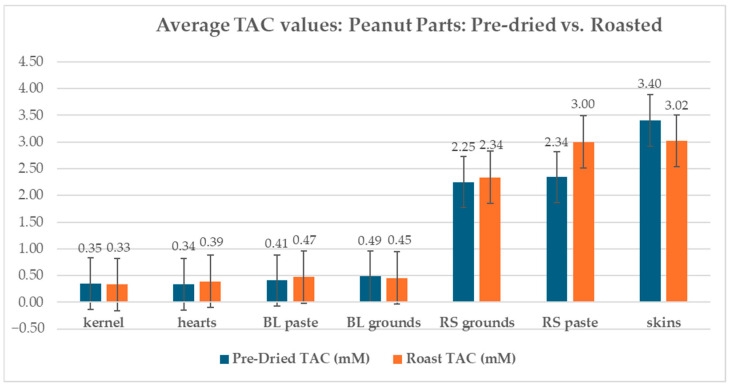
TAC of pre-dried and roasted peanut kernel, hearts, skins, blanched paste, blanched ground meal, red-skinned ground meal, and red-skinned paste.

Peanut skin revealed a TAC of 3.40 mM when pre-dried and 3.02 mM when roasted. Kernels showed a TAC of 0.35 mM when pre-dried and a TAC of 0.33 mM when roasted. Hearts showed a TAC of 0.34 mM when pre-dried and a TAC of 0.39 mM when roasted. Mass adjustment of the SE Jumbo runners (percent skin 2.94%) revealed that of the total TAC of roasted, red-skinned Southeast Jumbo Runner peanuts, 21.67% can be attributed to peanut skin and 78.33% can be attributed to a combination of kernel and heart (Figure 7).

### 2.2. Roasting Effect on TAC

Roasted samples revealed both increases and decreases when compared to the pre-dried samples of the same type. Peanut kernels showed a 5.47% decrease in TAC from 0.35 mM to 0.33 mM, while peanut skins showed a larger decrease of 11.26% from 3.40 mM to 3.02 mM when roasted. Concerning the three parts evaluated, skins, hearts, and kernels, roasting had the greatest effect on the TAC of peanut hearts, showing an increase of 15.88% from 0.34 mM to 0.39 mM. Blanched grounds showed a 6.58% decrease in TAC from 0.49 mM to 0.45 mM when roasted. Upon roasting, blanched peanut paste showed a 16.00% increase in TAC from 0.41 mM to 0.47 mM. Pre-dried, red-skinned peanuts (2.94 PS%) ground to a meal showed a 3.93% increase in TAC when roasted from 2.25 mM to 2.45 mM. Red-skinned paste showed a TAC increase of 28.08% from 2.34 mM to 3.00 mM when roasted.

### 2.3. Processing Effect on TAC in Peanuts

The designated processing stages were kernel, processed grounds, and processed pastes. Pre-dried kernels increased in TAC by 38.78% when processed into a meal. When pre-dried kernels were blanched and blended to a paste, the TAC revealed a smaller increase of 16.34%, from a TAC of 0.35 mM to 0.4070 mM. Roasted peanuts’ processing stages showed a kernel TAC of 0.33 mM, a blanched ground meal TAC of 0.45 mM, and a blanched paste TAC of 0.47 mM. Upon processing a meal, roasted peanuts showed a 37.15% increase in TAC and a 42.76% increase in TAC when processed from kernel to paste.

### 2.4. TAC Amount of Peanut Paste with Peanut Skin Supplementation

Blanched peanut paste had a TAC of 0.47 mM. Peanut paste with 1% PS had a TAC of 3.21 mM (an increase of 580.99%). Peanut paste with 3% PS had a TAC of 3.31 mM (an increase of 600.30%). Peanut paste with 5% PS had a TAC of 2.19 mM (an increase of 364.53%). Red-skinned peanuts had a TAC value of 3.00 mM (an increase of 535.52%). Commercially available “Unsalted Unblanched Peanut Butter” had a TAC of 2.59 mM (an increase of 448.66%).

### 2.5. Sensory

#### 2.5.1. Modified 10-Point Flavor Profile

**Figure 8:**
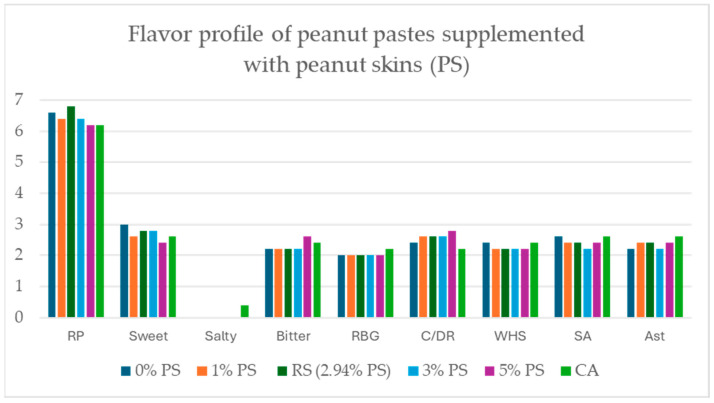
Modified flavor profile of peanut pastes supplemented with peanut skins (PS) at 0%, 1%, RS (2.94%), 3%, 5%, and Commercially Available “Unsalted Unblanched Peanut Butter” (CA). No significant changes of flavor profile were discovered among all peanut paste samples. Attribute abbreviations are RP=Roasted Peanut; RBG = Raw/Beany/Green; C/DR = Coffee/Dark Roast; WHS = Woody/Hulls/Skins; SA = Sweet Aromatic; Ast=Astringent.

ANOVA scores from panelists’ flavor analysis revealed no significant differences in flavor profile across all roasted peanut paste samples (0% PS, 1% PS, 3% PS, 5% PS, red-skinned, and commercially available “Unsalted Unblanched Peanut Butter”) (*p* < 0.05) (Figure 8).

#### 2.5.2. Structured Line Ranking

**Figure 9:**
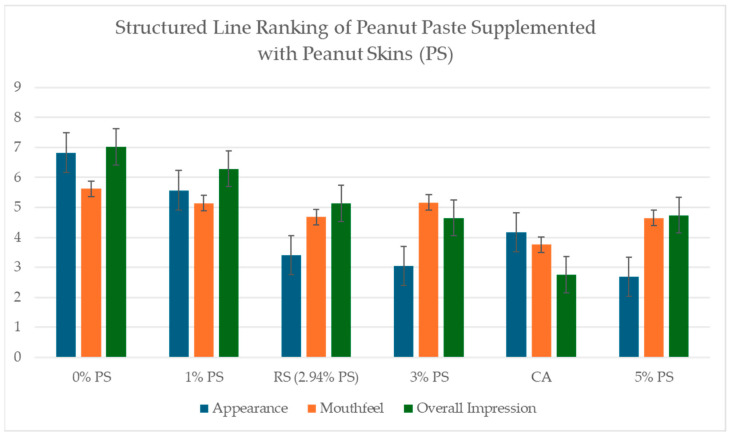
Structured line ranking scores of Appearance, Mouthfeel, and Overall Impression of peanut pastes supplemented with peanut skins (PS) at 0%, 1%, RS (2.94%), 3%, 5%, and Commercially Available “Unsalted Unblanched Peanut Butter” (CA). Significant differences (*p* > 0.05) were experienced in overall impression, with 5%PS scoring lower that 1% PS and the blind control (0% PS). Significant difference was seen most in appearance scores with 3% PS and 5% PS scoring lower than blind control, 1% and commercially available. No significant difference was experienced in mouthfeel.

Line ranking scores of appearance, mouthfeel, and overall impression were normalized by measuring the distance of the panelist response and dividing by 9.5. Normalized scores were analyzed through ANOVA testing. No significant differences in mouthfeel were reported by panelists (*p* < 0.05). Significant differences (*p* > 0.05) were seen in overall impression, with 5% PS scoring lower than 1% PS and the blind control (0% PS). A significant difference was seen most in appearance scores, with 3% PS and 5% PS scoring lower than the blind control and 1% commercially available (Figure 9).

#### 2.5.3. Hedonic Preference Test

Hedonic preferences generally showed an inversely proportional relation between the concentration of peanut skins and ranking placement. Exceptions to this relationship were seen twice. The red-skinned paste sample, having an approximate PS % of 2.94, was preferred less than the sample with 3% PS supplementation. The commercially available sample, which is estimated to have approximately 3% peanut skin concentration, ranked last in preference.

### 2.6. Principal Component Analyses

**Figure 10:**
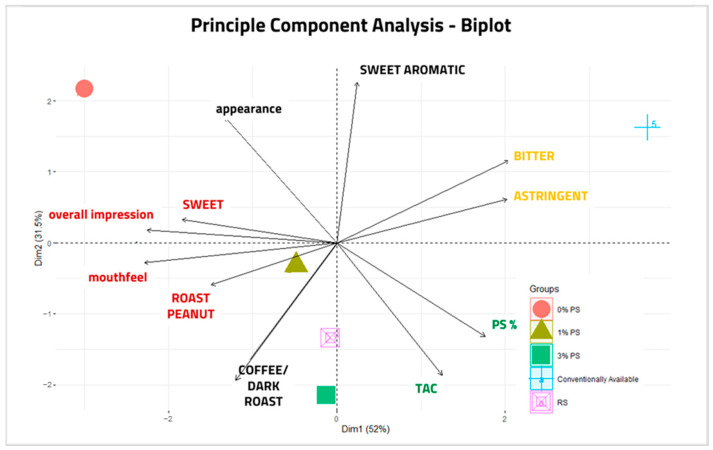
PCA-Biplot performed with mean values from sensory evaluations (excepting attributes of insufficient variation), TAC, and peanut skins (PS)% from paste samples: 0% PS, 1% PS, 3% PS, red skin (RS), and commercially available (CA). 5% formulation was excluded due to error of TAC value. Principle component analysis revealed correlations of “Sweet”, “Roast Peanut”, Overall Impression, and Mouthfeel; TAC and PS%; and Bitter and Astringent. Appearance and TAC; and “Astringent” and “Roast Peanut” were inversely correlated.

Principal component analyses (PCAs) are used to show correlations and reduce the complexity of data representing different qualities present in a sample set. A PCA was performed to assess clusters and sensory trends as they relate to TAC and percent skins present in peanut paste formulations (Figure 10). Sensory data containing more than two variations in response averages (“salt”, “woody/hull/skins”, and “sweet aromatic” were excluded due to lack of variability), TAC values, and PS percentages were analyzed. Due to the unexpectedly low value of a TAC of 5% PS, this sample was also excluded. The first two principal components, Dim1 and Dim2, explained 52% and 31.5% of the total variance, respectively. Three semi-gathered clusters revealed the following correlations: “Sweet”, overall impression, mouthfeel, and “Roast Peanut”; “Bitter” and “Astringent”; and PS% and TAC. Inverse correlations were also seen in direct proportions between TAC and appearance, and near inverse correlations were seen between “Roast Peanut” and the “bitter”/” astringent” cluster. Plotting samples in the PCA revealed that 1% PS, 3% PS, and RS correlated stronger than both 0% PS and commercially available samples. Commercially available and 0% PS were plotted in isolation from other samples, suggesting that they do not correlate with other samples.

## 3. Discussion

In this study, we report the biodiversity of seed coat colors that exist within the peanut germplasm, the relative AOC of peanut components, analysis of the impact of seed coat color on TAC, the TAC of skin-supplemented roasted peanut paste, and sensory analysis of skin supplementation in roasted peanut paste. Once the protocol was optimized for seed coats and kernels, the CuPRAC assay demonstrated repeatability and consistency across test samples, making the assay an effective baseline measurement tool for comparison.

Seed coat colors (white, tan, pink, red, purple, dark purple), available from the four common market types of peanuts (Spanish, Valencia, Runner, and Virginia) originating from a variety of locations, were separated into kernels and seed coats and then assayed for TAC. Analysis of the relative AOC of seed coats and kernels revealed that as anticipated, seed coats contain measurable AOC, but so do the kernels. The individual TAC values expressed as TE varied from 0.28 mM to 0.78 mM in mass-adjusted kernel + skin. The kernel antioxidant composition percentage ranged from 56.3 to 88.4%. The results suggest that not only does peanut skin offer a healthful benefit but there is also a boost in AOC from the consumption of roasted peanut kernels.

In comparisons between TAC and seed coat color, as anticipated, the white seed coats contained the lowest TAC on average but the dark purple skin samples did not have the highest TAC. Surprisingly, samples with pink or tan seed coats contained the highest relative amount of TAC. This result further reinforces the idea that darker seed coat color is not necessarily correlated with TAC. Also, the darkest color categories did not demonstrate the highest TAC, except in the Runner market type, which coincidentally had the fewest skin color options in the selected sample set selected from GRIN resources. All four market types demonstrate similar TAC, which is available in a variety of skin colors. However, not all market types include genotypes with all skin color options readily available. From this data set, several accessions were identified for seed increase to allow for further analytical analysis and genomic studies. Questions remain regarding specific antioxidant compounds, which can contribute to seed coat color.

An increase in TAC was seen when peanut skins were included in peanut paste formulations. The inclusion of peanut skin at formulations approximate to the natural percent skin of the peanut can increase TAC by four times (commercially available unblanched peanut butter) to an almost 6-fold increase (3% PS formulation). A lower-than-expected TAC value was seen in the 5% PS formulation. It is unclear why the less-than-expected antioxidant level was seen in the 5% PS supplementation sample. Further research is needed to determine the cause and contributing factors. Concerning samples with supplemental peanut skins added back to the peanut paste formulations, values of absorbance were consistent with the duplicate absorbances, but reproducibility was low within the same sample aliquot. The samples of red-skinned peanuts (peanuts that had been roasted and blended in the red-skinned phase), the commercially available, and the 0% PS supplementation did not see the same lack of reproducibility. It can, therefore, be suggested that pre-blending the peanut skin introduced a level of instability of antioxidant capacity. It may also be suggested that when peanut skins are blended simultaneously with peanut kernels, the oil in the peanut kernel may have an encapsulation effect on the antioxidants, which may have a protectant effect on the stability of antioxidant mechanisms.

Peanut skin supplementation in peanut paste did not negatively affect the sensory quality or appearance of samples up to the 5% PS formation. Differences in individual flavor attributes shown in Figure 8 were not statistically significant. From the PCA analysis of sensory traits and TAC shown in Figure 10, overall impression and desirable qualities of sweet taste, mouthfeel, and roast peanut flavor were positively correlated. TAC was directly associated with PS%, bitter, and coffee/dark roast flavor attributes. The bitter and dark roast attributes are characteristic of roasted peanut flavor and did not significantly affect the perception and ranking by the panelists up to 3% PS supplementation. Roasting and processing affect the TAC in different ways. A decrease in TAC was observed when isolated roasted skin was compared to pre-dried skin, while increases in TAC were observed in peanut hearts and peanut pastes when roasted. Additional sensory analysis and TAC measurement in samples produced from seed increase would be valuable to compare once suitable seed samples are available for destructive testing.

This study utilized the CuPRAC assay, while previous studies analyzing peanut skin supplementation have utilized the H-ORAC or Folin–Ciocalteu assays; therefore, the numeric results cannot be directly compared. The results do, however, show a similar increase in antioxidant capacity [45,46]. The results for sensory implications of peanut skin supplementation in peanut pastes confirm the results reported [42]. These results confirm that a 5% w/w supplementation of peanut skins scores lower in acceptability and appearance and that a peanut skin supplementation approximate to the natural percent skin of a peanut (3%) is more acceptable to the consumer [42].

## 4. Materials and Methods

The selection of GRIN samples was made with the criteria of sufficient data in the following categories: variety of color, cultivar/market type, North or South American origin, linoleate/oleate data availability, and maturity. An effort was made to obtain a sample of each main market type (Spanish, Valencia, Virginia, and Runner) in each primary seed coat color (purple, red, pink, tan, and white) with varied maturities (early, medium, late, or very late). An initial accession of 12 peanut samples was obtained from the GRIN database followed by an additional 42 samples from the GRIN database. Phenotypic images were obtained for all 54 samples (Figure S7a–e). Countries of origin represented in the accession include Guatemala, Paraguay, Bolivia, Ecuador, Brazil, Peru, Argentina, Ecuador, the United States, and Cuba.

**Figure 11:**
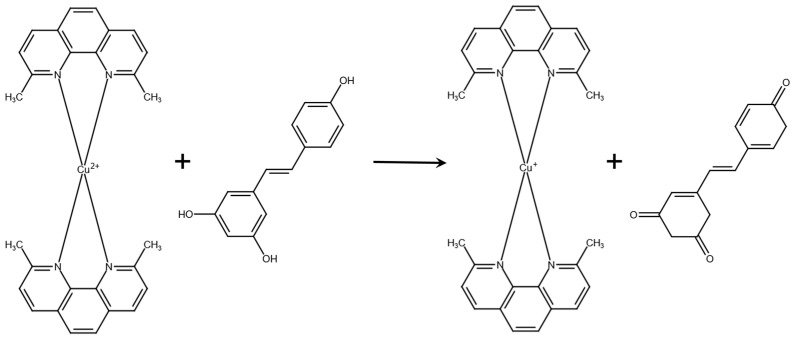
Cu(II)-Neocuproine in the presence of resveratrol is reduced to CuPRAC (Cu(I)-Neocuproine) as resveratrol is oxidized to its quinone.

There is not currently a standardized method to determine antioxidant capacity for all foods; instead, there are many ways to determine variable markers of antioxidant level or activity available. Various antioxidant quantification methods are used to analyze antioxidant activity in food. The benefits and deficiencies of antioxidant assays such as CuPRAC, ferric reduction in antioxidant power (FRAP), Folin–Ciocalteu, Oxygen Radical Absorption Capacity (ORAC), Hydroxyl Radical Antioxidant Capacity (HORAC), 2,2′-Azinobis-(3-ethylbenzothiazoline-6-sulfonic acid (ABTS), and 2,2-diphenyl-1-picrylhydrazyl (DPPH) are discussed in the literature [5,46]. The CuPRAC assay uses a copper (II) ion bonded to two neocuproine molecules (Figure 11). The CuPRAC assay was utilized in this study due to its well-documented application for physiological pH, the use of stable reagents, and the capability to scavenge lipophilic and hydrophilic antioxidants. 

The Abcam Total Antioxidant Capacity Kit (ab65329) includes reagents needed for running the colorimetric assay. Reagents are briefly centrifuged and equilibrated to room temperature before use. The Assay Diluent and protein mask are ready to use as supplied. To make the Cu2+ working solution, dilute 1 part of the Cu2+ reagent with 49 parts of Assay Diluent and mix well. The Trolox Standard is reconstituted in 20 μL of pure DMSO by vortexing in a vial followed by adding 980 μL of DI-H2O and mixing to generate a 1 mM Trolox Standard solution.

Peanut samples were massed into disruption tubes containing ceramic and zircon microbeads. A total of 1.5 mL ice-cold 1% PBS solution was added, and the samples were homogenized for 30 s using a Mini-Bead Beater 16 homogenizer from BioSpec Products (Bartletsville, OK). After homogenization, samples were incubated on ice for 10 min and then centrifuged for 3 m at 4 °C at top speed to remove insoluble materials. After centrifugation, samples were placed on ice. An aliquot of the sample was further diluted using ice-cold 1% PBS to ensure readings were within the standard curve range. A total of 50 µL diluted sample, 50 µL protein mask, and 100 µL Cu2+ working solution are added to each well.

For the standard curve, the Trolox Standard solution was aliquoted into sample wells according to the Abcam kit instructions. A total of 100 µL Cu2+ working solution was added to each standard well.

The plate was incubated 90 m at 20 °C at 300 rpm on an Eppendorf Thermomixer R. After incubation, measure the output on a microplate reader at OD 570 nm.

In February 2025, the TAC method calibration STD was calculated at 0.037177 with a % coefficient of variation of 8.797015.

To calculate the Trolox Equivalents, subtract the mean absorbance value of the blank (Standard #1) from all standard and sample readings. This is the corrected absorbance. Next, average the duplicate reading for each standard and sample. Plot the corrected absorbance values for each standard as a function of the final concentration of Trolox Equivalents. Draw the best smooth curve through these points to construct the standard curve. Calculate the trendline equation based on your standard curve data (use the equation that provides the most accurate fit). The concentration of Trolox Equivalents (nmol/μL or mM) in the test samples is calculated as follows:

$$Sample\;Total\;Antioxidant\;Capacity=\left(\frac{Ts}{Sv}\right)*\;D$$

where

Ts = TAC amount in the sample well calculated from a standard curve (nmol).
Sv = sample volume added in the sample wells (µL).
D = sample dilution factor.

Skin/kernel mass-adjusted calculations are as follows:

$$\frac{mass\;of\;dried\;kernel+skins-mass\;of\;dried\;kernel\;only}{mass\;of\;dried\;kernel+skins}*\;100=\%\;Skins$$

**Figure 12:**
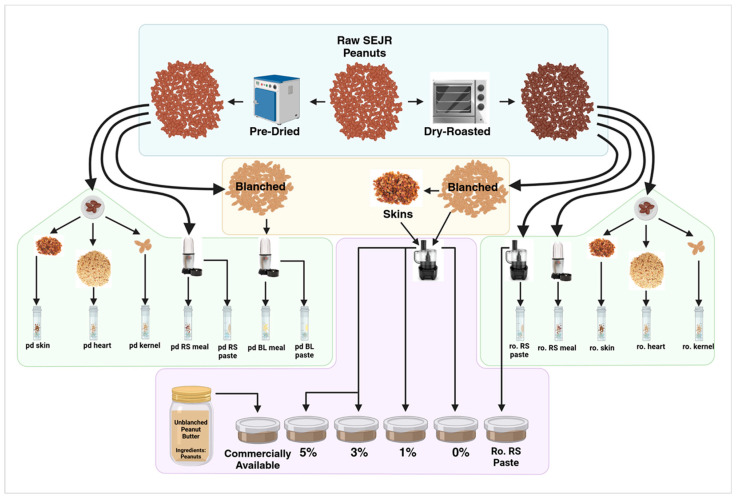
Schematic of TAC and sensory sample prep showing both pre-dried and roasted peanut parts including skins, hearts, and kernels, as well as redskin meals and skin-supplemented pastes.

Southeast Jumbo Runner peanuts were pre-dried or roasted following the Sensory Paste Preparation Protocol of BRL Analytical Services, LLC [47]. Approximately 300 g of raw peanut sample was placed in a single layer on a roasting pan and roasted at 177 °C in a Waring 500X convection oven to reference roast color. The sample is stirred occasionally and rotated through oven positions throughout roasting (i.e., the top, middle, and bottom of the oven). Roasted peanuts were cooled to ambient temperature, blanched if necessary, and then blended to paste using a food processor (Black + Decker model FP4200) (Figure 12).

Pre-dried peanuts were used to find the percent of skin and moisture loss after pre-drying. A composite sample (five red-skinned peanuts pre-dried and roasted) was selected and dissected into kernels, hearts, and skins. Pre-dried and roasted peanuts were then blanched or kept red-skinned and processed into rough meals and pastes. Pre-dried meals and pastes were blended in a small blender (Magic Bullet Express, Homeland Housewares, New York, NY, USA), and roasted pastes were blended in a food processor.

Roasted paste samples were supplemented with peanut skins at 0%, 1%, 3%, and 5%. A red-skinned paste made from unblanched, roasted peanuts, and a commercially available “Unblanched, Unsalted Peanut Butter” were also included. Each sample was given an identifier code unrelated to the peanut paste composition. All roasted paste samples were sent to an expert sensory panel at Lubbock Christian University and followed the sensory tasting protocols of the Biochemistry Research Lab [47]. Sensory panelists were compensated for their time by the Biochemistry Research Lab.

Sensory panelists scored each sample against a reference standard control paste and indicated scores on a supplied ballot. The sensory panel comprised highly trained, experienced expert panelists who received extensive initial training, a period of extensive provisional work prior to being an active panelist, and ongoing training at least annually. At the time of this study, each panelist had a minimum of eight consecutive years of experience serving on sensory panels. An average of five panelists’ scores were recorded for each sensory attribute. The sensory tasting station was supplied with appropriate ballots, writing utensils, clear cups for drinking, and red cups for disposing of individual samples after tasting. Panelists had access to unsalted crackers as a palate cleanser and fresh drinking water between samples [43].

Data analysis was conducted using a variety of statistical measures including an FAMD (factor analysis of mixed data), PCA (principal component analysis), MCA for categorial analysis (Multiple Correspondence Analysis), and box and whisker plots. The comparison of samples in this study required multiple methods to visualize the relationships between variables and determine the best method for the comparison of data. As the sample size of each unique sample was limited, as much as possible, the sample was represented by several kernels and seed coats from each genotype. More analytical testing of antioxidant composition and quantity as well as genomic analysis is being conducted in parallel studies requiring the scale up of seed from samples of interest. These studies are currently in progress.

## 5. Conclusions

Peanut blanching is a large industry that generates 750,000 metric tons of waste annually. This waste has a high valorization potential, as it contains a high total antioxidant capacity. The supplementation of peanut paste with peanut skin shows minimal changes in sensory experience and beneficial increases in TAC. Although differences in flavor scores were not significant, there was a difference in appearance and mouthfeel as PS% increased. The impact on the sensory experience may be minimized if peanut skin supplementation occurs in peanut butter or other food products already containing inclusions, such as sweeteners or flavor enhancers. Additionally, peanut skins may have added value in other food products for antimicrobial properties as well as antioxidant capacity. It should be noted, however, that caution should be taken when supplementing peanut skin, as there can be issues related to allergenicity. Products that do not normally contain peanuts could serve as an allergen hazard if supplemented with peanut skins without clear product communication.

The results from this study suggest that more investigation is needed into the antioxidant composition of kernels and seed coats. Parallel investigation, extracting, and isolating components for further analysis is ongoing. Given the results with the TAC of the kernels themselves, additional work is needed to quantify major and minor components corresponding to AOC. The kernels themselves may offer a measurable and impactful benefit without the addition of peanut skins. Further studies should look at specific phytosterol components of peanut paste with and without peanut skin inclusions by more advanced analysis methods such as gas chromatography–mass spectrometry and liquid chromatography–mass spectrometry. Additionally, the effect of processing and roasting on antioxidant capacity should be analyzed. Specifically, the processing of peanut skins and the physical manipulation’s effects on TAC should be assessed.

All samples provided by GRIN were grown under similar conditions and with inputs and irrigation according to best practices for seed development. This preparation provides a baseline set of samples for comparison. Differences in location, water availability, soil nutrition, and biotic and abiotic stress were not variables in this study. The impact of environmental factors will be an important aspect to consider. The CuPRAC assay method will allow for a comparison of TAC between specific genotypes grown in different conditions, providing another reason for generating additional seeds from the collection for further investigation.

In order for additional studies on the impacts of increasing genetic diversity of AOC within peanut breeding populations to occur, additional quantities of seeds are necessary. Collaborating peanut scientists are currently increasing seed quantities for further analytical studies as well as potential investigations on bioavailability, digestibility, and relationships to other macronutrients contributing to the AOC provided by the consumption of peanuts.

## Figures and Tables

**Table 1:** Mass-adjusted Total Antioxidant Capacity of GRIN subset (n = 54) as determined by commercially available CuPRAC assay. Samples were assayed as seed coat and kernel fractions separately, then mM TAC was calculated by mass-adjusting the components according to the calculated percent skins for each individual sample.

Mass-Adjusted Total Antioxidant Capacity as mM Trolox Equivalents													
		Skin		Kernel		Total			Skin		Kernel		Total
ID	Sample	TAC (mM)	Mass Adj	TAC (mM)	Mass Adj	S + K	ID	Sample	TAC (mM)	Mass Adj	TAC (mM)	Mass Adj	S + K
1	PI 196619	3.31	0.13	0.64	0.62	0.74	28	PI 336980	5.12	0.14	0.29	0.28	0.42
2	PI 196644	3.13	0.11	0.51	0.49	0.60	29	PI 337343	4.36	0.11	0.33	0.32	0.43
3	PI 196658	3.47	0.14	0.59	0.56	0.70	30	PI 337405	4.01	0.12	0.29	0.28	0.41
4	PI 268750	3.04	0.07	0.21	0.20	0.28	31	PI 337410	3.77	0.15	0.31	0.30	0.44
5	PI 270818	3.24	0.12	0.62	0.59	0.71	32	PI 337431	3.02	0.11	0.31	0.30	0.40
6	PI 306227	4.24	0.10	0.21	0.20	0.30	33	PI 365550	4.29	0.12	0.32	0.31	0.43
7	PI 331294	2.72	0.08	0.45	0.44	0.52	34	PI 403736	3.70	0.09	0.32	0.31	0.40
8	PI 342666	2.93	0.07	0.54	0.53	0.60	35	PI 403798	5.43	0.15	0.32	0.31	0.46
9	PI 596800	3.04	0.08	0.32	0.31	0.39	36	PI 408743	2.38	0.06	0.32	0.31	0.37
10	PI 602156	2.82	0.11	0.67	0.65	0.75	37	PI 501997	4.45	0.12	0.31	0.30	0.41
11	PI 493511	2.99	0.07	0.29	0.29	0.36	38	PI 561688	2.57	0.06	0.34	0.33	0.39
12	PI 576615	3.32	0.10	0.70	0.68	0.78	39	PI 565458	8.63	0.22	0.32	0.32	0.54
13	PI 152108	3.30	0.10	0.30	0.29	0.39	40	PI 602350	6.90	0.21	0.36	0.35	0.55
14	PI 161315	3.76	0.10	0.36	0.35	0.45	41	PI 602525	2.77	0.06	0.34	0.33	0.39
15	PI 162532	4.82	0.15	0.36	0.35	0.49	42	PI 628604	4.79	0.12	0.36	0.35	0.47
16	PI 162659	2.82	0.09	0.32	0.31	0.40	43	PI 628612	5.99	0.17	0.31	0.30	0.47
17	PI 162882	3.04	0.08	0.38	0.37	0.45	44	PI 475980	5.46	0.12	0.32	0.32	0.44
18	PI 259707	6.28	0.19	0.32	0.31	0.50	45	PI 476039	7.60	0.25	0.34	0.33	0.58
19	PI 259710	5.57	0.15	0.30	0.29	0.44	46	PI 493329	5.73	0.14	0.34	0.33	0.48
20	PI 259721	5.19	0.15	0.30	0.30	0.44	47	PI 497586	8.61	0.21	0.31	0.30	0.51
21	PI 261985	5.16	0.14	0.30	0.30	0.44	48	PI 561221	2.81	0.06	0.32	0.32	0.38
22	PI 261993	5.00	0.12	0.31	0.30	0.42	49	PI 540833	4.77	0.12	0.34	0.33	0.45
23	PI 262035	4.45	0.12	0.29	0.28	0.40	50	PI 628577	3.44	0.07	0.38	0.37	0.45
24	PI 329225	2.51	0.05	0.32	0.31	0.36	51	PI 494000	6.00	0.21	0.35	0.34	0.55
25	PI 331299	5.72	0.15	0.33	0.32	0.48	52	PI 494008	4.78	0.18	0.36	0.35	0.53
26	PI 331313	6.05	0.22	0.35	0.34	0.56	53	PI 494027	5.06	0.16	0.31	0.30	0.46
27	PI 331326	2.39	0.05	0.31	0.30	0.35	54	PI 494056	4.28	0.08	0.32	0.31	0.39

## Data Availability

The original contributions presented in this study are included in the article/Supplementary Materials. Further inquiries can be directed to the corresponding author(s).

## Supplementary Materials

The following supporting information can be downloaded at https://www.mdpi.com/article/10.3390/ijms26135990/s1, References [48,49] are cited in the Supplementary Materials.

## Abbreviations

The following abbreviations are used in this manuscript:

GRIN	Germplasm Resource Information Network
LDL	Low-density lipoprotein
LDL/HDL	Low-density lipoprotein/high-density lipoprotein
ROS	Reactive oxygen species
USDA-FAS	United States Department of Agriculture Foreign Agricultural Service
TPC	Total phenolic content
TAC	Total antioxidant capacity
FRAP	Ferric-ion-reducing antioxidant power
AOC	Antioxidant content
TE	Trolox equivalent
FAMD	Factor analysis of mixed data
CuPRAC	CuPric-reducing antioxidant capacity
RP	Roast peanut
RBG	Raw, beany, green
C/DR	Coffee, dark roast
WHS	Woody, hulls, skins
SA	Sweet aromatic
AST	Astringent
PS	Peanut skin
RS	Red-skinned
PCA	Principal component analysis
ORAC	Oxygen radical absorption capacity
HORAC	Hydroxyl radical antioxidant capacity
PBS	Phosphate-buffered saline
STD	Standard deviation
