# Supplementary material for: Total Antioxidant Capacity of Arachis hypogaea Seed Kernels and Coats: An Analytical and Sensory Investigation
Source: Int J Mol Sci. 2025 Jun 22;26(13):5990. doi: 10.3390/ijms26135990 (PMC12250297; doi:10.3390/ijms26135990)
Supplement: Supplementary file 1 [file ijms-26-05990-s001.zip › ijms-3645533-supplementary.pdf]

Supplementary Materials

Catechins+PACs

**Monomers to trimer**	**Method**	**Source**
(+)-catechin	HPLS and ESI-MS	http://dx.doi.org/10.1016/j.chroma.2014.06.027
(−)-epicatechin	HPLS and ESI-MS	http://dx.doi.org/10.1016/j.chroma.2014.06.027
PAC B type dimers	HPLS and ESI-MS	http://dx.doi.org/10.1016/j.chroma.2014.06.027
PAC A-type dimers	HPLS and ESI-MS	http://dx.doi.org/10.1016/j.chroma.2014.06.027
	HRMS–MS/MSafterincubationwithCaco-2cells	https://doi.org/10.1111/1750-3841.70018
	UHPLC–Q-Orbitrap HRMSanalysis.	https://doi.org/10.1111/1750-3841.70018
PAC A-type trimers with 1 A-bond	HPLS and ESI-MS	http://dx.doi.org/10.1016/j.chroma.2014.06.027
PAC A-type trimers with 2 A-bonds	HPLS and ESI-MS	http://dx.doi.org/10.1016/j.chroma.2014.06.027
B-type trimers	HPLS and ESI-MS	http://dx.doi.org/10.1016/j.chroma.2014.06.027
	UHPLC–Q-Orbitrap HRMSanalysis.	https://doi.org/10.1111/1750-3841.70018
PAC A-type tetramer [4(epi)cat-3A]	UHPLC–Q-Orbitrap HRMSanalysis.	https://doi.org/10.1111/1750-3841.70018
PACA/B-type tetramer [4(epi)cat-2A]	UHPLC–Q-Orbitrap HRMSanalysis.	https://doi.org/10.1111/1750-3841.70018
PACA/B-type tetramer [4(epi)cat-2A]	UHPLC–Q-Orbitrap HRMSanalysis.	https://doi.org/10.1111/1750-3841.70018
PACA/B-type tetramer [4(epi)cat-1A]	UHPLC–Q-Orbitrap HRMSanalysis.	https://doi.org/10.1111/1750-3841.70018
PACA/B-type tetramer [4(epi)cat-1A]	UHPLC–Q-Orbitrap HRMSanalysis.	https://doi.org/10.1111/1750-3841.70018
PACA/B-type tetramer [3(epi)cat-1Gal 2A]	UHPLC–Q-Orbitrap HRMSanalysis.	https://doi.org/10.1111/1750-3841.70018
PACA-type trimer [3(epi)cat-2A]	UHPLC–Q-Orbitrap HRMSanalysis.	https://doi.org/10.1111/1750-3841.70018
ProcyanidinA1, PACA-typedimer [(epi)cat-A (epi)cat]	UHPLC–Q-Orbitrap HRMSanalysis.	https://doi.org/10.1111/1750-3841.70018
PACA-typedimer [(epi)gal-A (epi)cat]	UHPLC–Q-Orbitrap HRMSanalysis.	https://doi.org/10.1111/1750-3841.70018
PACA-typedimer [1(epi)cat 1(epi)gal-1A]	UHPLC–Q-Orbitrap HRMSanalysis.	https://doi.org/10.1111/1750-3841.70018
(Epi)gallocatechin gallate	UHPLC–Q-Orbitrap HRMSanalysis.	https://doi.org/10.1111/1750-3841.70018
(Epi)catechin gallate	UHPLC–Q-Orbitrap HRMSanalysis.	https://doi.org/10.1111/1750-3841.70018
**PACs containing luteolin or kaempferol units**	**Method**	**Source**
A-type trimer containing a luteolin or kaempferol unit	HPLS and ESI-MS	http://dx.doi.org/10.1016/j.chroma.2014.06.027
PAC dimer containing a luteolin or kaempferol unit	HPLS and ESI-MS	http://dx.doi.org/10.1016/j.chroma.2014.06.027
**Propelargonidins**	**Method**	**Source**
PAC dimer with an (epi)afzelechin top unit connected to a subsequent (epi)catechin unit through an A-type linkage	HPLS and ESI-MS	http://dx.doi.org/10.1016/j.chroma.2014.06.027
PAC trimer with an (epi)afzelechin top unit connected to a subsequent (epi)catechin unit through an A-type linkage	HPLS and ESI-MS	http://dx.doi.org/10.1016/j.chroma.2014.06.027
**Prodelphinidin and prorobinetidin**	**Method**	**Source**
prodelphinidin A-type dimer	HPLS and ESI-MS	http://dx.doi.org/10.1016/j.chroma.2014.06.027
	UHPLC–Q-Orbitrap HRMSanalysis.	https://doi.org/10.1111/1750-3841.70018
prorobinetidin dimer	HPLS and ESI-MS	http://dx.doi.org/10.1016/j.chroma.2014.06.027
	UHPLC–Q-Orbitrap HRMSanalysis.	https://doi.org/10.1111/1750-3841.70018

Figure S1. Catechins and PACs found in peanut skins [48, 49]

Stilbenes, Stillbenoids

**Stilbenes**	**Method**	**Source**
trans resveratrol	HPLS and ESI-MS	http://dx.doi.org/10.1016/j.chroma.2014.06.027
trans-piceatannol	HPLS and ESI-MS	http://dx.doi.org/10.1016/j.chroma.2014.06.027
**Stillbenoid**	**Method**	**Source**
Piceid	HPLS and ESI-MS	http://dx.doi.org/10.1016/j.chroma.2014.06.027
trans-Resveratrol	HPLS and ESI-MS	http://dx.doi.org/10.1016/j.chroma.2014.06.027
Quercetin	HPLS and ESI-MS	http://dx.doi.org/10.1016/j.chroma.2014.06.027

Figure S2. Stilbenes and stillbenoids found in peanut skins [48].

Flavenoids

Isoflavones	Method	Source
3′,5,7 trihydroxyiso flavone-4′ methoxy-3′-O-β glucopyranoside	UHPLC–Q-Orbitrap HRMSanalysis.	https://doi.org/10.1111/1750-3841.70018
	HPLS and ESI-MS	http://dx.doi.org/10.1016/j.chroma.2014.06.027
biochanin A	HPLS and ESI-MS	http://dx.doi.org/10.1016/j.chroma.2014.06.027
	UHPLC–Q-Orbitrap HRMSanalysis.	https://doi.org/10.1111/1750-3841.70018
Dihydroxymethox yprenylisoflavone	UHPLC–Q-Orbitrap HRMSanalysis.	https://doi.org/10.1111/1750-3841.70018
Methylated prenylated flavonoid	UHPLC–Q-Orbitrap HRMSanalysis.	https://doi.org/10.1111/1750-3841.70018
**Flavanones**	**Method**	**Source**
Homoeriodictyol	HPLS and ESI-MS	http://dx.doi.org/10.1016/j.chroma.2014.06.027
eriodictyol	HPLS and ESI-MS	http://dx.doi.org/10.1016/j.chroma.2014.06.027
	UHPLC–Q-Orbitrap HRMSanalysis.	https://doi.org/10.1111/1750-3841.70018
Taxifolin	UHPLC–Q-Orbitrap HRMSanalysis.	https://doi.org/10.1111/1750-3841.70018
Manniflavanone	UHPLC–Q-Orbitrap HRMSanalysis.	https://doi.org/10.1111/1750-3841.70018
**Flavones**	**Method**	**Source**
Apigenin O-hexoside	UHPLC–Q-Orbitrap HRMSanalysis.	https://doi.org/10.1111/1750-3841.70018
Luteolinor Kaempferol O-hexoside	UHPLC–Q-Orbitrap HRMSanalysis.	https://doi.org/10.1111/1750-3841.70018
Luteolinor kaempferol O-deoxyhexosyl hexoside	UHPLC–Q-Orbitrap HRMSanalysis.	https://doi.org/10.1111/1750-3841.70018
luteolin	HPLS and ESI-MS	http://dx.doi.org/10.1016/j.chroma.2014.06.027
diosmetin	HPLS and ESI-MS	http://dx.doi.org/10.1016/j.chroma.2014.06.027
cirsiliol	HPLS and ESI-MS	http://dx.doi.org/10.1016/j.chroma.2014.06.027
**Flavonols and flavanonols**	**Method**	**Source**
quercetin	HPLS and ESI-MS	http://dx.doi.org/10.1016/j.chroma.2014.06.027
Quercetin glucuronide	HPLS and ESI-MS	http://dx.doi.org/10.1016/j.chroma.2014.06.027
Quercetin O-hexoside	UHPLC–Q-Orbitrap HRMSanalysis.	https://doi.org/10.1111/1750-3841.70018
Quercetinmethyl ether	UHPLC–Q-Orbitrap HRMSanalysis.	https://doi.org/10.1111/1750-3841.70018
isorhamnetin	HPLS and ESI-MS	http://dx.doi.org/10.1016/j.chroma.2014.06.027
	HRMS–MS/MSafterincubationwithCaco-2cells.	https://doi.org/10.1111/1750-3841.70018
Isorhamnetin O-pentoside	UHPLC–Q-Orbitrap HRMSanalysis.	https://doi.org/10.1111/1750-3841.70018
Isorhamnetin acetyl glucoside	HPLS and ESI-MS	http://dx.doi.org/10.1016/j.chroma.2014.06.027
dihydrorobinetin dimethyl ether	HPLS and ESI-MS	http://dx.doi.org/10.1016/j.chroma.2014.06.027
3,4-dimethyl dihydroquercetiN	HPLS and ESI-MS	http://dx.doi.org/10.1016/j.chroma.2014.06.027
Rutin	UHPLC–Q-Orbitrap HRMSanalysis.	https://doi.org/10.1111/1750-3841.70018
**Biflavonoids**	**Method**	**Source**
daphnodorin H-type	HPLS and ESI-MS	http://dx.doi.org/10.1016/j.chroma.2014.06.027
morelloflavone	HPLS and ESI-MS	http://dx.doi.org/10.1016/j.chroma.2014.06.027

Figure S3. Flavenoids found in peanut skins [49, 48].

Free Phenolic Acids+Esters

**Hydroxybenzoic acids**	**Method**	**Source**
Protocatechuic acid	HPLS and ESI-MS	http://dx.doi.org/10.1016/j.chroma.2014.06.027
Dihydroxybenzoic acid	UHPLC–Q-Orbitrap HRMSanalysis.	https://doi.org/10.1111/1750-3841.70018
Hydroxybenzoyl hexose	UHPLC–Q-Orbitrap HRMSanalysis.	https://doi.org/10.1111/1750-3841.70018
p-Hydroxybenzoic acid	HPLC	DOI: 10.5897/AJB12.1389

**Hydroxycinnamicacids**	**Method**	**Source**
Coutaric acid	UHPLC–Q-Orbitrap HRMSanalysis.	https://doi.org/10.1111/1750-3841.70018
Dihydroxycinnamic acidhexoside	UHPLC–Q-Orbitrap HRMSanalysis.	https://doi.org/10.1111/1750-3841.70018
Caftaricacid	UHPLC–Q-Orbitrap HRMSanalysis.	https://doi.org/10.1111/1750-3841.70018
cis-Caffeicacid	UHPLC–Q-Orbitrap HRMSanalysis.	https://doi.org/10.1111/1750-3841.70018
Chicoricacid	UHPLC–Q-Orbitrap HRMSanalysis.	https://doi.org/10.1111/1750-3841.70018
Fertaricacid	UHPLC–Q-Orbitrap HRMSanalysis.	https://doi.org/10.1111/1750-3841.70018

**Caffeic acid**	**Method**	**Source**
Isoferulic acid	HRMS–MS/MSafterincubationwithC aco-2cells.	https://doi.org/10.1111/1750-3841.70018
caffeoyltartaric acid (caftaric acid),	HPLS and ESI-MS	http://dx.doi.org/10.1016/j.chroma.2014.06.027
dicaffeoyltar taric acid (chicoric acid)	HPLS and ESI-MS	http://dx.doi.org/10.1016/j.chroma.2014.06.027

**Coumaric acids**	**Method**	**Source**
p-Coumaric acid	HPLS and ESI-MS	http://dx.doi.org/10.1016/j.chroma.2014.06.027
	HPLC	DOI: 10.5897/AJB12.1389
o-coumaric acid	HPLS and ESI-MS	http://dx.doi.org/10.1016/j.chroma.2014.06.027
cis-coutaric acid (p-coumaroyltartaric acid)	HPLS and ESI-MS	http://dx.doi.org/10.1016/j.chroma.2014.06.027
trans-coutaric acid	HPLS and ESI-MS	http://dx.doi.org/10.1016/j.chroma.2014.06.027
p-coumaroyl-O-pentoside	HPLS and ESI-MS	http://dx.doi.org/10.1016/j.chroma.2014.06.027
o-coumaroyl-O-pentoside	HPLS and ESI-MS	http://dx.doi.org/10.1016/j.chroma.2014.06.027
p-comaroylnictotinoyltartaric acid	HPLS and ESI-MS	http://dx.doi.org/10.1016/j.chroma.2014.06.027
di-p-coumaroyltartaric acid	HPLS and ESI-MS	http://dx.doi.org/10.1016/j.chroma.2014.06.027
p-coumaroyltartaric acid ether-linked formononetin	HPLS and ESI-MS	http://dx.doi.org/10.1016/j.chroma.2014.06.027
trans-resveratrol,	HPLS and ESI-MS	http://dx.doi.org/10.1016/j.chroma.2014.06.027
piceid	HPLS and ESI-MS	http://dx.doi.org/10.1016/j.chroma.2014.06.027
p-coumaroyl-p-hydroxybenzoyltartaric acid	HPLS and ESI-MS	http://dx.doi.org/10.1016/j.chroma.2014.06.027
p-coumaroylvanilloyltartaric acid	HPLS and ESI-MS	http://dx.doi.org/10.1016/j.chroma.2014.06.027
p-Coumaroylcaffeoyltartaric acid	HPLS and ESI-MS	http://dx.doi.org/10.1016/j.chroma.2014.06.027
p-coumaroylsinapoyltartaric acid	HPLS and ESI-MS	http://dx.doi.org/10.1016/j.chroma.2014.06.027
p-coumaroylferuloyltartaric acid	HPLS and ESI-MS	http://dx.doi.org/10.1016/j.chroma.2014.06.027

**Ferulic acid and its esters**	**Method**	**Source**
Ferulic acid	HPLC	DOI: 10.5897/AJB12.1389
feruloyltar taric acid (fertaric acid)	HPLS and ESI-MS	http://dx.doi.org/10.1016/j.chroma.2014.06.027
feruloyl aspartate	HPLS and ESI-MS	http://dx.doi.org/10.1016/j.chroma.2014.06.027
caffeoylferulic acid	HPLS and ESI-MS	http://dx.doi.org/10.1016/j.chroma.2014.06.027
Feruloylcaffeoyltartaric acid	HPLS and ESI-MS	http://dx.doi.org/10.1016/j.chroma.2014.06.027
Isoferulic acid	HRMS–MS/MSafterincubationwithC aco-2cells.	https://doi.org/10.1111/1750-3841.70018

**Coumarin**	**Method**	**Source**
dihydroxycoumarin	HPLS and ESI-MS	http://dx.doi.org/10.1016/j.chroma.2014.06.027
Vanillic acid	HPLC	DOI: 10.5897/AJB12.1389
Sinapinic acid	HPLC	DOI: 10.5897/AJB12.1389
Syringic acid	HPLC	DOI: 10.5897/AJB12.1389

Figure S4. Free phenolic acids and esters found in peanut skins [48, 49].

Preference Ranking Ballot

Please rank the samples according to preference:

Most preferred

1 Sample ID: ______________________________

2 Sample ID: ______________________________

3 Sample ID: ______________________________

4 Sample ID: ______________________________

5 Sample ID: ______________________________

6 Sample ID: ______________________________

7 Sample ID: ______________________________

Least Preferred

Figure S5. Sensory panel preference ranking ballot.

Descriptive Flavor and Hedonic Line Ranking Ballot

PEANUT PASTE BALLOT

Sample ID: ______________________

Panelist: ____________________ Date: ____________________

SAMPLE MUST BE ROASTED CORRECTLY. IF SAMPLE IS OVER/UNDER ROASTED, NOTIFIY SAMPLE PREP PERSONNEL AND NOTE IN COMMENTS.

	INTENSITY									
ATTRIBUTE	10	9	8	7	6	5	4	3	2	0/1
Roasted PEANUT										
Sweet										
Salty										
Bitter										
Raw/Beany Green										
Coffee/Dark Roast										
Woody/Hull/Skin										
Sweet Aromatic										
Astringent										

Please rate overall likeability by drawing a vertical line on the scale:

Dislike Extremely — Neither like nor dislike — Like Extremely

Appearance

Mouthfeel

Overall impression

Please provide any additional notes regarding the flavor and sensory experience for this sample:

Figure S6. Sensory panel descriptive and structured hedonic line ranking ballot.

Phenotypic images of select GRIN germplasm samples

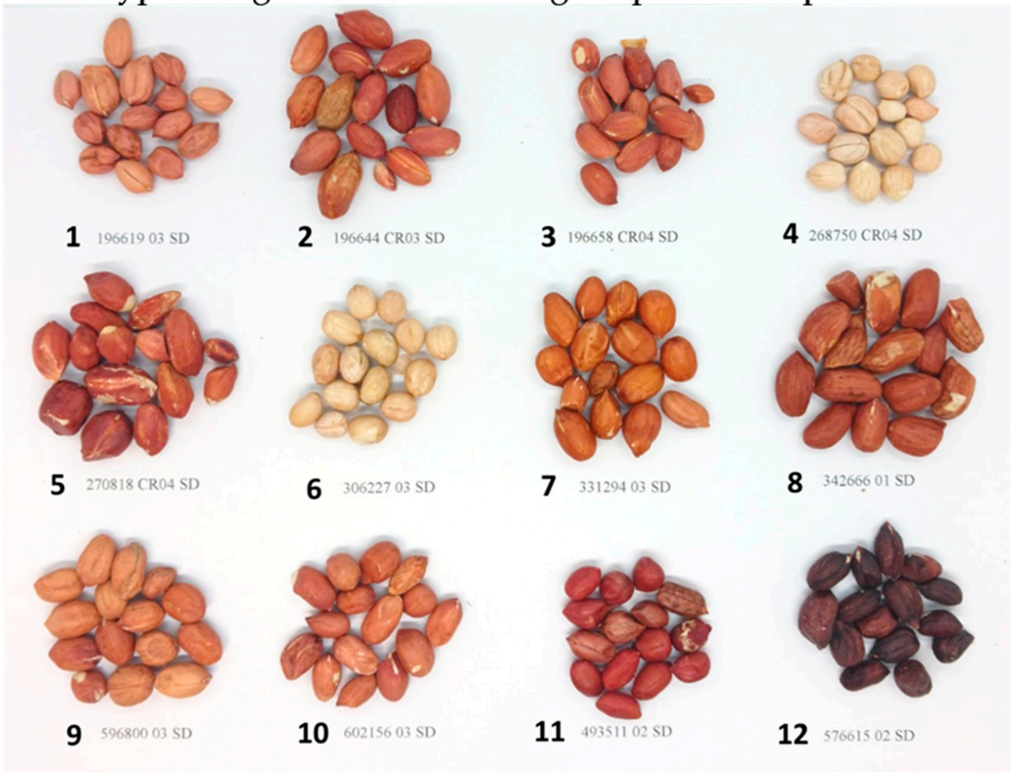

Figure S7a. Phenotypic images of select GRIN samples 1-12.

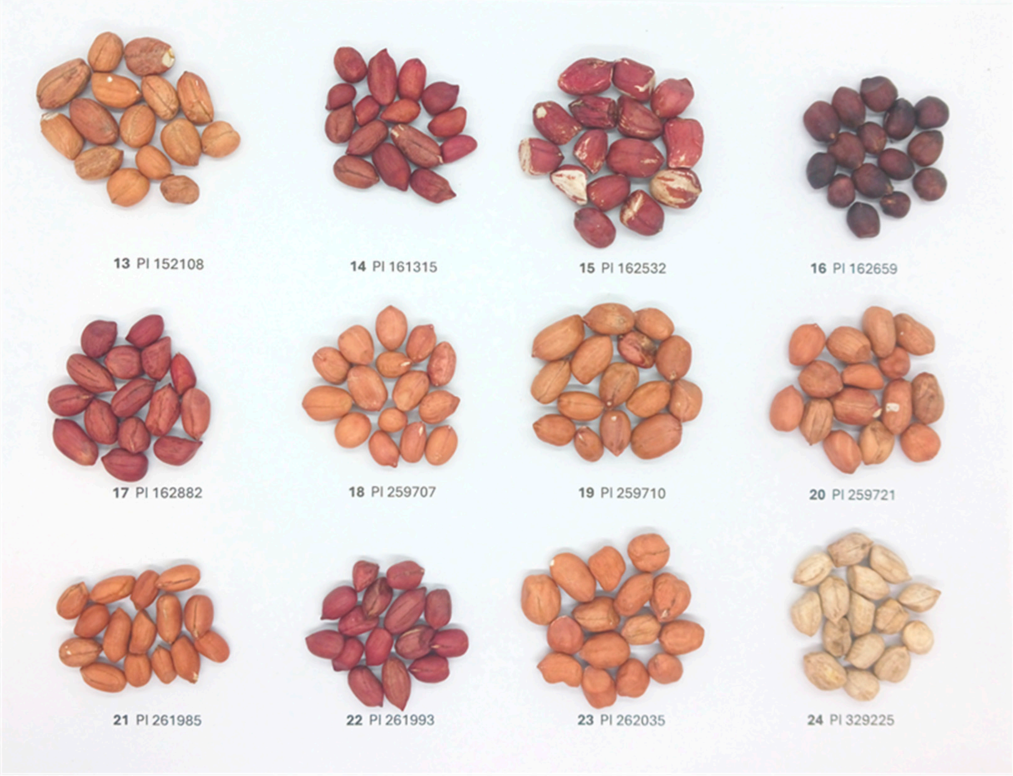

Figure S7b. Phenotypic images of select GRIN samples 13-24.

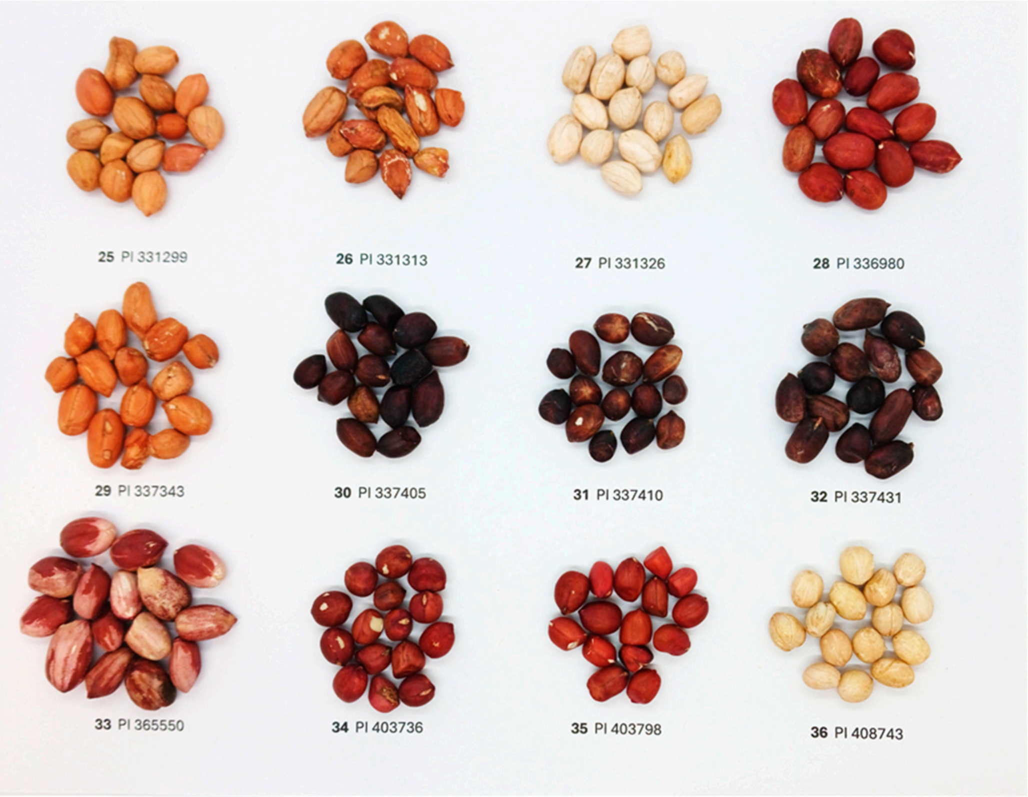

Figure S7c. Phenotypic images of select GRIN samples 25-36.

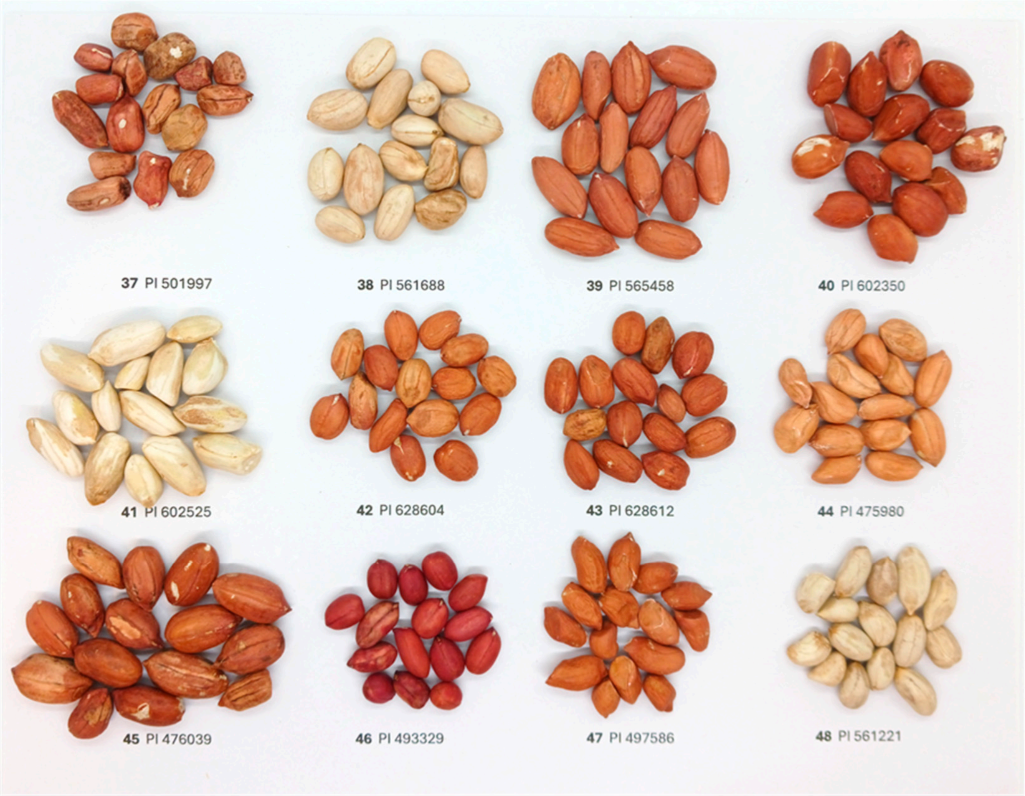

Figure S7d. Phenotypic images of select GRIN samples 37-48.

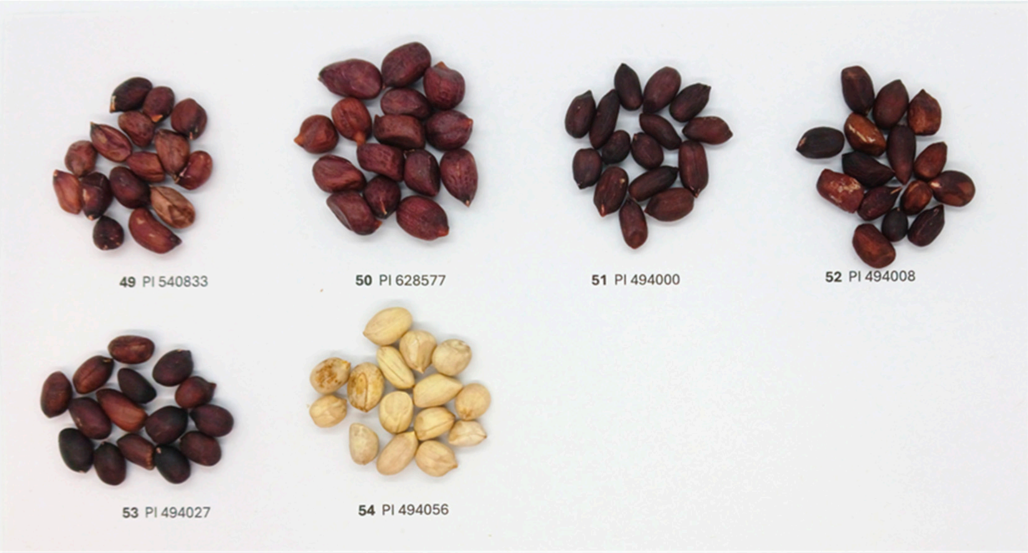

Figure S7e. Phenotypic images of select GRIN samples 37-54.
